# Preparation of Ni/Cu composite nanowires

**DOI:** 10.3762/bjnano.6.130

**Published:** 2015-06-05

**Authors:** Hu Wang, Xiaoyu Li, Ming Li, Kenan Xie, Li Liao

**Affiliations:** 1 School of Chemical Engineering, Sichuan University, Chengdu 610065, PR China

**Keywords:** copper nanoparticles, nickel nanowires, Ni/Cu composite nanowires

## Abstract

Ni/Cu composite nanowires were synthesized in an aqueous solution for the first time. The synthetic process consisted of two steps. Firstly, pure nickel nanowires were prepared through chemical reduction in solution under a magnetic field. Secondly, copper was reduced on the surface of the nickel nanowires, during which Ni/Cu composite nanowires with an average length of 80 µm and diameter of about 200 nm were synthesized. The products were characterized by XRD, SEM and TEM. The method has notable advantages: It is template-free, inexpensive, easy-to-operate, and it only needs a short reaction time, which makes it suitable for large-scale preparation.

## Introduction

In recent years, the catalytic hydrogenation of CO_2_ to light hydrocarbons has been recognized as one of the most effective and economical ways to fix and utilize a large amount of anthropogenic CO_2_ [1–3]. Nickel and copper, which are extensively applied in industry, are efficient hydrogenation catalysts both with regard to conversion and selectivity of CO_2_ hydrogenation [4–6]. Nanoparticles of metals possess the advantages of a small grain diameter and a high specific surface area [7–9]. However, the active region on the surface cannot be completely used because of aggregation of the particles [10–13]. In contrast, one-dimensional nanostructures especially nanowires of metallic materials have the advantage of a bigger active region and more interspaces owing to their unique morphology with a space grid structure [14–19]. To the best of our knowledge, no research about preparing composite nanowires of nickel and copper has been reported to date. It is, however, of great significance to develop a new one-dimensional material containing both nickel and copper.

In this research, a facile route to synthesize composite nanowires with copper nanoparticles on the surface of nickel nanowires, named Ni/Cu composite nanowires, was developed for the first time. The synthetic process consisted of two steps. Firstly, pure nickel nanowires were prepared in solution without template by applying a weak external magnetic field. Secondly, the obtained nickel nanowires were used as reductant to reduce Cu^2+^ to synthesize Ni/Cu composite nanowires. Compared with nickel or copper nanowires of the same diameter, the Ni/Cu composite nanowires are expected to perform better in CO_2_ hydrogenation because their surface consists of a great number of small particles, which increases the specific surface area of the nanowires.

## Experimental

In the experiments, all the reagents are analytical grade and are used as received. At first, nickel nanowires were prepared in solution under a magnetic field through a facile method [20]. In a typical process, 0.16 g NiSO_4_·6H_2_O (0.01 mol·L^−1^) and 0.18 g C_6_H_5_Na_3_O_7_·2H_2_O (0.01 mol·L^−1^) were dissolved in 60 mL deionized water. The pH value of the solution was adjusted to 12.5 and the temperature of the solution was kept at 80 °C. Hydrazine hydrate (N_2_H_4_·H_2_O) was added as reducing agent. The molar ratio of N_2_H_4_·H_2_O/NiSO_4_·6H_2_O was 2:1. Secondly, the prepared pure nickel nanowires were added into 60 mL of CuSO_4_ solution (the molar ratio of Cu/Ni was 1:2) at 80 °C. The resulting products were collected, rinsed three times with deionized water and ethanol respectively. Ultimately, the synthesized products were dried in vacuum for further testing and applications.

The morphology of the samples was observed by field emission scanning electron microscopy (FE-SEM, JSM-7500F, JEOL) operating at 10 kV accelerating voltage, and energy dispersive spectroscopy (EDS) analysis was performed with the spectrometer attached to the same SEM. The composition and crystallographic properties of the products were analyzed by X-ray diffraction (XRD, Philips, X’pert) in the range from 20 to 85° with a scanning rate of 0.02°/s. Transmission electron microscopy (TEM) images were obtained with a JEOL JEM-100CX TEM.

## Results and Discussion

**Figure 1:**
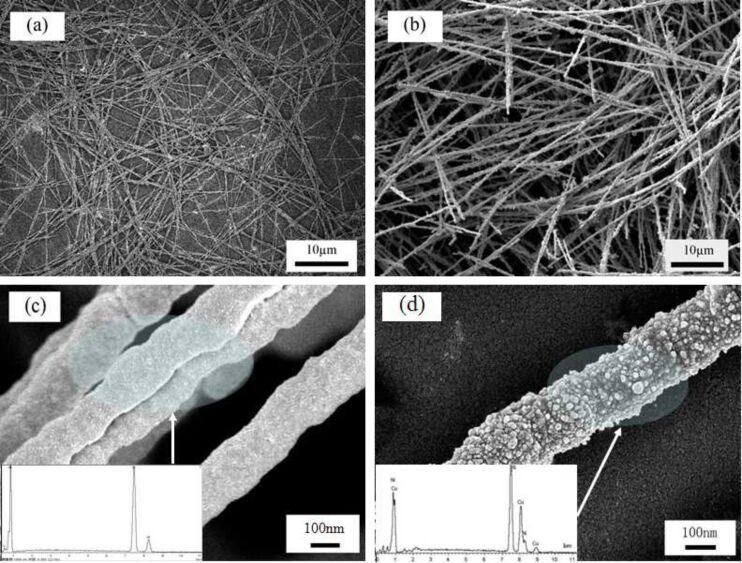
SEM images of Ni nanowires under different magnification (a,c) and Ni/Cu composite nanowires under different magnification (b,d), insets (c,d) are EDS images of Ni nanowires and Ni/Cu composite nanowires.

The SEM image of synthesized Ni nanowires before being used as a special template is shown in Figure 1a. Figure 1b shows the SEM image of the prepared Ni/Cu nanowires under optimal conditions that can be achieved at present. Uniform Ni/Cu composite nanowires with an average size of approximately 200 nm in diameter and 80 µm in length were obtained. Furthermore, no isolated spherical Ni or Cu particles are observed in Figure 1b. By contrasting Figure 1a and Figure 1b, no obvious difference in morphology can be found.

SEM images of Ni nanowires and Ni/Cu composite nanowires at a high magnification are shown in Figure 1c and Figure 1d, respectively. It is obvious that Ni nanowires (Figure 1c) of about 200 nm in diameter with a relatively smooth surface were obtained before being used as template. Figure 1d shows the surface morphology of the Ni/Cu composite nanowires, from which a straight clubbed nanowire could be observed with its surface coated by plenty of small particles. We assume these small grains to be copper particles. It can be concluded from the difference of the surface morphology between Ni nanowires and Ni/Cu composite nanowires that copper ions were reduced by nickel, which resulted in copper particles deposited on the surface of the Ni nanowires. Copper particles were nucleating and growing fast, while nickel decreased on the surface. Intensive ultrasonication for 30 min did not disintegrate the Ni/Cu composite nanowires and no dispersive particles could be observed, which implies the mechanical stability of Ni/Cu composite nanowires and confirms that they are not merely a loose aggregate of spherical nanoparticles. The EDS graph of the synthesized Ni/Cu composite nanowires in a typical experiment is shown in Figure 1d. The analysis data show that the contents of nickel and copper were 62.34 and 37.32%, respectively, which indicated the high purity of prepared Ni/Cu composite nanowires with few impurities and confirmed our assumption.

**Figure 2:**
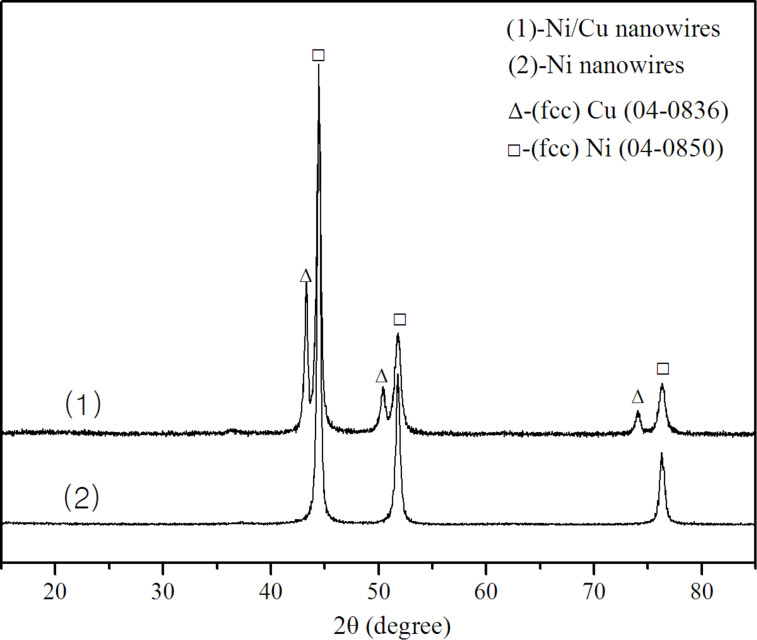
XRD patterns of Ni/Cu nanowires (1) and Ni nanowires (2).

Figure 2 shows the corresponding XRD spectrum for the resultant wires. It can be well indexed with the reflections of face-centered cubic Ni (PDF standard cards, JCPDS 04-0850, space group *Fm*−3*m*) and face-centered cubic Cu (PDF standard cards, JCPDS 04-0836, space group *Fm*−3*m*) without impurity peaks, which is consistent with the EDS data given in Figure 1d. Three characteristic peaks of face-centered cubic Ni (2θ = 44.56, 51.92 and 75.44°) correspond to Miller indices (111), (200) and (220), respectively. The cell parameter is *a* = 3.545 Å. Three characteristic peaks of face-centered cubic Cu (2θ = 43.38, 50.58, 74.20°) correspond to Miller indices (111), (200) and (220), respectively. The cell parameter is *a* = 3.615 Å. It can be easily observed that the characteristic peak intensity of nickel is stronger than that of copper since the reduced copper was simply on the surface of nickel nanowires. From this, it is unequivocal that the grains on the surface of Ni nanowires are copper particles.

**Figure 3:**
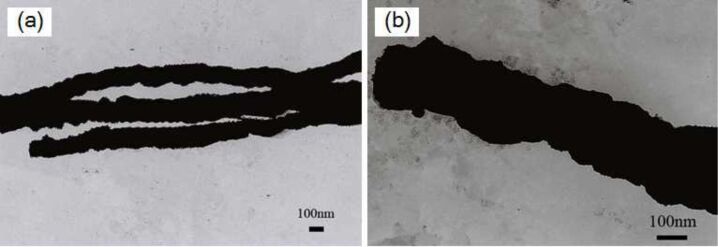
TEM images of prepared Ni nanowires (a) and Ni/Cu nanowires (b).

Figure 3 shows the TEM images of Ni nanowires (a) and Ni/Cu composite nanowires (b). It can be observed from Figure 3a that nickel particles are assembled into a solid linear structure under an external magnetic field, which correspond with our previous research [7,20]. It is obvious that the surface of the Ni/Cu composite nanowires is compact from the Figure 3b, which further confirmed that the copper grains are not simply attached to the surface of Ni nanowires.

## Conclusion

Polycrystalline Ni/Cu composite nanowires of approximately 200 nm in diameter and 80 µm in length were synthesized for the first time through a new method combining the self-assembly of nickel particles under an external magnetic field and the reduction of Cu^2+^ on the surface of prepared nickel nanowires. It is found that an external magnetic field can make nickel particles self-assemble in one dimension, leading to the formation of polycrystalline nickel nanowires. Copper ions were then reduced to tiny particles on the surface of the prepared nickel nanowires. The present study provides a simple method to prepare uniform Ni/Cu composite nanowires in large scale with great potential for industrial applications, especially in the field of CO_2_ hydrogenation.
